# Involvement of End Users in the Development of Serious Games for Health Care Professions Education: Systematic Descriptive Review

**DOI:** 10.2196/28650

**Published:** 2021-08-19

**Authors:** Marc-André Maheu-Cadotte, Véronique Dubé, Sylvie Cossette, Alexandra Lapierre, Guillaume Fontaine, Marie-France Deschênes, Patrick Lavoie

**Affiliations:** 1 Faculty of Nursing Université de Montréal Montreal, QC Canada; 2 Research Center Centre hospitalier de l'Université de Montréal Montreal, QC Canada; 3 Research Center Montreal Heart Institute Montreal, QC Canada

**Keywords:** game-based learning, health professions education, participatory design, systematic review, user-centered design, serious games, game development, end users, education

## Abstract

**Background:**

On the basis of ethical and methodological arguments, numerous calls have been made to increase the involvement of end users in the development of serious games (SGs). Involving end users in the development process is considered a way to give them power and control over educational software that is designed for them. It can also help identify areas for improvement in the design of SGs and improve their efficacy in targeted learning outcomes. However, no recognized guidelines or frameworks exist to guide end users’ involvement in SG development.

**Objective:**

The aim of this study is to describe how end users are involved in the development of SGs for health care professions education.

**Methods:**

We examined the literature presenting the development of 45 SGs that had reached the stage of efficacy evaluation in randomized trials. One author performed data extraction using an ad hoc form based on a design and development framework for SGs. Data were then coded and synthesized on the basis of similarities. The coding scheme was refined iteratively with the involvement of a second author. Results are presented using frequencies and percentages.

**Results:**

End users’ involvement was mentioned in the development of 21 of 45 SGs. The number of end users involved ranged from 12 to 36. End users were often involved in answering specific concerns that arose during the SG design (n=6) or in testing a prototype (n=12). In many cases, researchers solicited input from end users regarding the goals to reach (n=10) or the functional esthetics of the SGs (n=7). Most researchers used self-reported questionnaires (n=7).

**Conclusions:**

Researchers mentioned end users’ involvement in the development of less than half of the identified SGs, and this involvement was also poorly described. These findings represent significant limitations to evaluating the impact of the involvement of end users on the efficacy of SGs and in making recommendations regarding their involvement.

## Introduction

Serious games (SGs) are video games designed with a primary educational purpose [1]. SGs are based on the premise that learners who experience high levels of engagement and motivation during an educational experience can achieve better learning outcomes [2]. Thus, SGs combine design elements such as goals, rewards, and narrative events, that are likely to evoke positive emotions in learners (eg, joy, surprise), capture and sustain their attention, and fuel their desire to play to offer an engaging and motivating learning experience [2]. In health care professions education, developing an SG can be a long, complex, and expensive undertaking, as the input of a team of several actors including content experts and game designers is required [3,4]. Olszewski and Wolbrink [3] proposed a three-stage development framework to promote the efficiency of this process and efficacy of an SG. In the first design stage, the team must establish the learning objectives and map learners’ experience in the SG. This entails defining the goals, feedback, and rewards as well as the narrative and esthetics that will bring the virtual world to life and allow interactions [2]. During the second programing stage, SG design elements are gradually combined into one or several prototypes. In the third testing stage, the team tests these prototypes and suggests modifications to the design of the SG. Throughout the development process, the team must pay close attention to various design principles to ensure that learners remain motivated and engaged and that the SG is effective for learning [2,4]. For example, the knowledge and skills needed to meet the goals presented in the SG should match the learners’ knowledge and skill level. If learners perceive the goals as being too easy or difficult, they may become bored or stressed [5].

The involvement of end users (in this case, the health care professionals and students for whom the SG is intended) in the SG development process may help ensure that these design principles are followed and that the SG offers an engaging and motivating learning experience [6]. Researchers [7,8] have described different roles or levels of involvement for end users in the development of SGs: they can be consulted about their learning needs and design preferences in the first design stage, or they can provide feedback and answers to specific concerns that arise later during the second programing stage. Some end users may be involved at the third stage of prototype testing, while others may be co-designers if they lead or contribute substantially to the development process.

However, involving end users could increase the complexity of the SG development process and, by extension, the cost and time needed. Assessing end users’ learning needs and design preferences before establishing learning objectives and mapping their experience, or having end users test prototypes, are additional steps that require further resources and planning [7,9-11] with no guarantee of cost-effectiveness according to current evidence. For example, in their systematic review, DeSmet et al [8] found that health games developed with patients as co-designers were not more effective than those in which patients were not involved in development. Along with authors of previous reviews and studies, they underlined a paucity of data on how end users are selected to participate in SG development, the methods used to elicit their input, the elements on which their input is solicited, and the extent to which their input is integrated into the SG [8,11,12]. These data could allow researchers and developers to consider the involvement of end users based on others’ experience in this field.

Thus, in the absence of evidence, guidelines, or a design framework to specifically guide end users’ involvement, this systematic review aimed to describe end users’ involvement in the development of SGs for health care professions education. Specifically, we sought to answer the following questions:

1. What criteria are used to select end users in the development of SGs?
2. How are end users involved in the development of SGs?
3. What SG design elements are assessed and modified following end users’ involvement?

## Methods

### Review Design

This study was a descriptive review of end users' involvement (concept) in the development of SGs (context) for health care professionals and students (population). Descriptive reviews allow the identification of trends in a representative sample of published literature regarding prespecified methodological or theoretical elements [13]. This descriptive review builds on the methods used and results found in a previous systematic review aimed at evaluating the efficacy of SGs in health care professions education [14,15].

For this review, “end users’ involvement” was considered an umbrella term for inviting health care professionals and students to contribute to the design or refinement of an SG in any of the three stages of the development process prior to efficacy evaluation [3]. Health care professionals and students with any level of education (from undergraduate to postgraduate education, continuing education) or from any clinical setting were considered. However, SGs for patients were not considered. All SGs that were included aimed to improve learning outcomes (eg, knowledge, skills, attitudes, behaviors) related to various clinical situations or topics.

### Reference Identification and Selection

For a previous systematic review [14], we developed a search strategy to identify randomized controlled trials (RCTs), evaluating the efficacy of SGs among health care professionals and students. The search strategy combined keywords and index terms related to health care professions (eg, *nurses*, *medical* students), SGs (eg, *game-based learning*, *educational game*), and learning outcomes (eg, *knowledge acquisition*, *skill development*). On May 26, 2020, we searched six bibliographical databases: Cumulative Index of Nursing and Allied Health (EBSCO), EMBASE (OVID), ERIC (ProQuest), PsycINFO (APA PsycNET), PubMed (NCBI), and Web of Science—SCI and SSCI (ISI – Thomson Scientific). Two review authors performed the reference selection independently, identifying 45 SGs whose efficacy had been evaluated in 46 published RCTs. Further details regarding the search strategy and selection process for this previous review are published elsewhere [14-16]. The complete search strategy for all bibliographical databases is also presented in Multimedia Appendix 1.

For the current review, we focused on all development work prior to these 46 RCTs. As evaluating the efficacy of an intervention represents one of the last stages in its development [17-19], we considered that SGs that had been the object of RCTs had gone beyond the prototype programing and testing phases [3]. Thus, including only SGs that had been the object of RCTs allowed us to be confident that their development was complete as well as the end users’ involvement in it. One review author performed a backward reference search in the reference lists of the 46 RCTs to identify prior work that described the development of the SGs. When the name of an SG was provided, this review author also performed hand searches in Google to identify additional work describing its development. We included all types of work regarding the development of the SGs (eg, qualitative or quantitative empirical research, discussion on the development process) and all types of reporting (eg, conference abstract, poster, journal article, web page). A second review author also independently identified work related to the development of 8 SGs chosen randomly (18% of the 45 included SGs). This was to ensure that all relevant work was included. In medical record reviews, an independent audit of at least 10% of the sample is frequently recommended [20]; however, as no additional references could be identified for these 8 SGs, it was deemed satisfactory.

### Data Extraction and Synthesis

The unit of analysis was the included SGs. All documents related to a single SG were considered concurrently to describe the characteristics of end users’ involvement during the development of a particular SG. Thus, all frequency counts are based on the number of SGs rather than the number of papers included in this review.

Using an ad hoc data extraction grid based on the review aims and questions, one reviewer extracted all excerpts regarding end users’ involvement in SG development and categorized them according to the research questions:

- What criteria were used to select end users: end users’ involvement (ie, reported or not), number of end users involved, and eligibility criteria
- How were end users involved: what role was assigned to end users in the development of SGs? The roles were as follows: (1) as consultants at the onset of design, to share their learning needs or design preferences; (2) as consultants during design, to provide feedback and answers to specific concerns; (3) as prototype testers, toward the end of development; and (4) as co-designers throughout development, as a regular member of the team [3,7,8]. We also extracted the methods used to elicit end users’ input (eg, individual interviews, think-aloud methods).
- What SG design elements were assessed and modified following end users’ involvement: elements for which end-user input was elicited (see Table 1) and its influence on SG development
- Researchers’ views and recommendations on end users’ involvement

We coded data of the elements for which end users’ input was elicited and how their input was integrated into the SG (ie, how the SG was modified following end-user input) based on the SG design framework by Alexiou and Schippers [2]. We further synthesized them using an inductive approach based on data similarities. A second review author independently performed data extraction and coding. We refined the coding scheme until no difference from the results of the first reviewer was noted. This occurred after data extraction and coding were performed in a random sample of 8/45 SGs (18%).The results are presented narratively and by using descriptive statistics (frequencies and percentages) when appropriate.

**Table 1:** Elements in the serious game design framework by Alexiou and Schippers [2].

Element	Definition
Esthetics	Audio and visual elements that allow learners to perceive a harmonious and coherent virtual world (hedonic esthetics, eg, beauty or realism of the audiovisual rendering, background music) and to interact with the serious game (functional esthetics, eg, the user interface)
Narrative	The perspective through which learners explore the virtual world (protagonist), the figures that inhabit this world with whom learners can interact (secondary characters), and the situations that arise from learners’ actions and mark the evolution of this world (narrative events, eg, new game levels)
Game mechanics	What learners are expected to achieve in the serious game (goals), what they receive for doing so (rewards, eg, points, badges), and the help provided to facilitate their progression (feedback)

## Results

**Figure 1:**
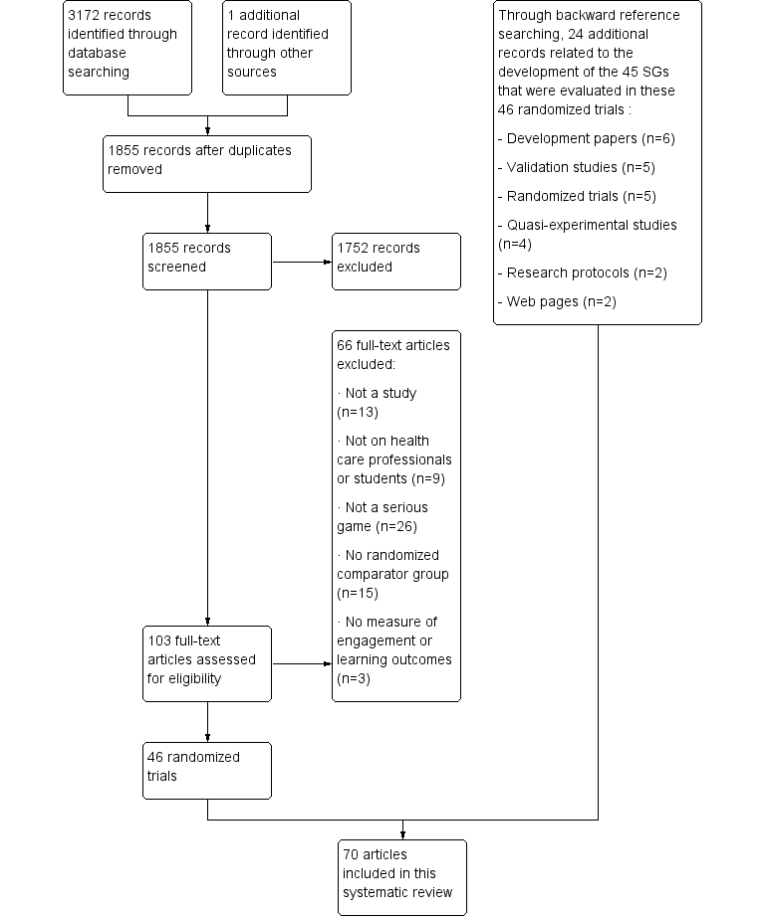
PRISMA (Preferred Reporting Items for Systematic Reviews and Meta-Analyses) flow diagram. SG: serious game.

Development of the 45 SGs was described in 70 papers. Figure 1 presents the PRISMA (Preferred Reporting Items for Systematic Reviews and Meta-Analyses) flow diagram for paper selection. End users’ involvement in the development process was explicitly mentioned for 21/45 SGs (47%; see Multimedia Appendix 2). The following sections present results regarding end user involvement in the development of these 21 SGs.

### What Selection Criteria Were Used to Select End Users in the Development of SGs?

The number of end users involved was reported for 9 of the 21 SGs (43%) and ranged from 12 to 36, with a median of 27 (IQR: 16) [21-29]. For 3/21 SGs (14%), the number of end users was defined by convenience: all readily accessible individuals were approached, and those who agreed to participate were enrolled [21,23,26]. No justification was found for the number of end users involved in the remaining 6/21 SGs (29%) [22,24,25,27-29].

Eligibility criteria for end-user selection were reported for the development of 1 of the 21 SGs (5%). Researchers selected an equal number of men and women with varying degrees of experience in gaming, but they did not provide a rationale for that decision [24].

### How Were End Users Involved During the Development of SGs?

We identified the role given to end users in the development process of 17/21 SGs (81%) [21-37]. Table 2 reports the end users’ roles in SG development and the methods used to elicit their input.

**Table 2:** End users’ roles in the development of serious games (N=21).

Role and methods used to elicit input^a^			Value, n (%)		References
**Consultant at the onset of the design stage**			2 (10)		[35,37]
	Undescribed questionnaire	1 (5)		[35]	
	Not reported	1 (5)		[37]	
**Consultant later in the design stage**			6 (29)		[25,28,29,32,36,37]
	Multiple-choice questionnaire	2 (10)		[25,29]	
	Focus group	1 (5)		[32]	
	Not reported	3 (14)		[28,36,37]	
**Prototype tester**			12 (57)		[21-24,26-28,30,33-35,37]
	Ad hoc Likert scales with written comments	3 (14)		[22,23,34]	
	Adaptation of the System Usability Scale	1 (5)		[24]	
	Undescribed questionnaire	1 (5)		[35]	
	Think-aloud method	1 (5)		[24]	
	Recording of in-game interactions	1 (5)		[24]	
Co-designers			1 (5)		[31]
Unclear			4 (19)		[38-41]

^a^End users had more than one role in the development of 3 serious games [28,35,37].

As Table 2 shows, end users were more often prototype testers (12/21, 57%), and their input was frequently elicited through questionnaires (7/21, 33%). Details regarding the content of these questionnaires and their development process were rarely provided (4/21, 19%). For one SG, authors used the System Usability Scale [42] with additional items regarding its ease of use and alignment with end users’ design preferences [24,43]. The System Usability Scale consists of 10 statements regarding the ease and speed of use of software, and end users are asked to express their level of agreement with each statement on a 5-point scale. Besides questionnaires, researchers also recorded end users’ interactions with a prototype of the SG and asked them to think aloud during their gaming experience. Based on the effect on end users’ experience with the SG, each interaction was then classified as either positive, neutral, or negative [24].

Only 1 of the 21 SGs (5%) was developed with end users as co-designers who oversaw the development of the clinical content [31]. This SG aimed to improve nurses’ confidence and skills in teaching the correct inhaler technique to patients. With these learning objectives in mind, a group of nurses developed the clinical content, which consisted of a description of seven steps to be followed during the self-administration of inhaled medication. The rest of the development team then developed the narrative of the SG around these seven steps.

### What SG Design Elements Were Assessed and Modified Following End Users’ Involvement?

Elements for which end-user input was elicited were reported for 15/21 SGs (71%) [21-26,28-31,34-37,39]. Researchers also stated that they modified 10 of the 21 SGs (48%) according to end-user input [23-25,27,30,33-35,37,39]; however, they detailed the modifications for only 5 SGs (24%) [24,25,27,35,39]. Table 3 reports the results regarding end users’ input in SG design. End users’ input was most frequently elicited regarding the goals of the SGs (10/21, 48%); their input regarding hedonic esthetics (2/21, 10%) and narratives (1/21, 5%) rarely received focus.

**Table 3:** End users’ input on serious game design.

Serious game design element	Value, n (%)	References	Aspects for which input was elicited	Modifications made to the serious game
Functional esthetics	7 (33)	[21-24,28,37]	Instructions for interacting with the serious game, interface clarity, and ease of use	More emphasis on visual cues in the virtual environment, reduction of the written material on the screen, addition of highlights and shadows to facilitate visualization of the cursor, and correction of technical glitches
Hedonic esthetics	2 (10)	[24,35]	Volume of the background music	Addition of options to switch off or decrease the volume of the background music
Protagonist and secondary characters	1 (5)	[22]	Length of the dialogues between the protagonist and secondary characters	Modifications not detailed
Narrative events	0 (0)	N/A^a^	N/A	N/A
Goals	10 (48)	[22-25,29,31,35-37,39]	Level of challenge and validity of the learning content	Tailoring of the level of challenge
Feedback	3 (14)	[24,26,34]	Feedback complexity and how it helped end users situate their progression	Addition of a progression bar to provide further feedback on end users’ progression
Rewards	0 (0)	N/A	N/A	N/A

^a^N/A: not applicable.

## Discussion

### Principal Results

Our review of 70 references indicated that end users were involved in the development of less than half the 45 SGs in health care professions education. They most often took the role of prototype testers during the later stages of SG development, and they were rarely involved as co-designers or consultants at the onset of development. In addition, researchers often used questionnaires to elicit end-user input. Other methods such as focus groups and individual interviews were rarely used. The level of challenge and functional esthetics were the aspects of SGs for which end users’ input was most frequently elicited.

### Comparison With Prior Work

Several criteria could be used to select end users in the development of SGs. In this review, criteria were mentioned for only one SG and focused on gender and gaming experience [24]. Garber et al [44] underlined that current evidence does not clearly support gender differences in learning preferences. Indeed, suggested gender differences in SGs relate to gameplay preferences (ie, competition for men and collaboration for women) and their perceived educational value (ie, higher in men than in women) [44,45]. However, in health care professions education, once the SGs reached the stage of efficacy evaluation, gender-based analyses have yet to reveal significant differences in learning outcomes [46-48]. Thus, the extent to which SG design should be informed by end users’ gender to improve its efficacy remains unknown. Diehl et al [24] mentioned gaming experience as another criterion for the selection of end users, noting that users with a small or large amount of gaming experience provided the richest input. Similarly, Boeker et al [22] reported that end users with the least amount of gaming experience had the most issues in their interactions with the SG. Given the limited evidence regarding criteria for end user selection, we suggest that researchers should include all end users without considering predetermined characteristics. We encourage them to report on differences in the input of end users based on gender, gaming experience, or other characteristics that could have played a role in the input obtained and in the result of their involvement.

The number of end users involved in SG development ranged from 12 to 36, and these numbers seemed to be mostly based on convenience, as the available end users were approached. Current sample size estimation approaches that are not focused on statistical power only seem suited to testing SG prototypes, as the numbers they suggest can still be considerable [49,50]. However, as the development and refinement of an SG is highly iterative, and many versions of the design of the game are proposed during the first development stages [3], including several end users at the onset of development can represent a challenge. In the broader fields of intervention development and participatory research, authors have suggested that establishing a dialogue with a small number of end users during the initial development stages—aiming for depth rather than breadth—is more important than obtaining a large sample size [17,51]. This suggestion is consistent with qualitative methods such as individual interviews or focus group discussions. However, the use of qualitative methods was described only twice, and only once before the testing of an SG prototype [24,32]. Thus, we suggest that researchers explore methods to elicit end users’ input that are suited for smaller samples, such as qualitative methods. This approach may help researchers acquire a rich understanding of end users’ perspectives on the design of an SG.

The results also elucidate the gap between eliciting and integrating end-user input into the SG design. Few researchers described the changes made to SGs following end-user involvement, and none discussed their decision-making process. Kelly [52] argued that considering end users’ input can be uncomfortable for researchers and, therefore, Kelly recommends that the researchers attempt to find a compromise between making all the decisions themselves and relinquishing all forms of decisions to end users. It has been suggested that not all end users’ input should be directly integrated into an SG and that experts in the field should review what could potentially improve the efficacy of an SG [9,53]. Further research is needed to identify what elements of end users’ input are the most valuable, and at what stage of the development process; thus, researchers should describe their rationale for eliciting end users’ input and their decision-making process for integrating this input.

In this review, we did not identify literature (published or accessible on the web) on the development of most SGs that were the object of an RCT. However, in papers detailing the results of RCTs, we found many instances of authors referring to previous unpublished studies related to the development of their SGs [28,31,39]. This could point to the existence of literature on development that researchers or editors of scientific journals did not consider suitable for publication [54,55]. We argue that sharing this type of experience may prove valuable for planning future SG developments. Further, as current publications of work related to the development of SGs often focus on end users as prototype testers, researchers should consider publishing their experience with end users as consultants, especially in the first stages of SG development or as co-designers. In this review, we found that end users often served the needs of researchers and developers either to answer their concerns or to test a prototype through a fixed protocol. This limits end users’ contribution to designing SGs as well as researchers’ ability to potentially substantially change the design of an SG once it has reached the testing phase.

### Limitations

The strengths of this descriptive review are that it entailed a comprehensive literature search to ensure that all SGs that had been the object of an RCT were found. The selection process was conducted independently and in pairs to ensure that all relevant papers were included; however, SGs that had not been the object of an RCT were not considered in this review. If the development of these SGs were still in progress, only a partial portrait of their development would have been provided. Other limitations include the data extraction process, which was conducted by a single review author for most SGs. This was judged adequate, as the aim of this work was descriptive and no efficacy data were extracted. Finally, as underlined in the discussion, this review was limited to work available on the internet.

### Conclusions

Considering that end users’ involvement was poorly described in the SGs under review, we suggest that researchers publish information on the nature of end-user involvement, including the characteristics of the end users selected, content of the instruments used to elicit their input, modifications made to the SGs based on end-user input, and lessons learned throughout the development process. As few researchers reported end users’ involvement in the initial development, those opting for this type of involvement should consider sharing their views on the process. Moreover, researchers should consider involving end users with varying levels of gaming experience and combining different methods to elicit their input to gain further insights into issues that may undermine the efficacy of SGs.

## Appendix

Multimedia Appendix 1 All search strategies.

Multimedia Appendix 2 Key elements of end users' involvement in the development of serious games in health care professions education.

## Abbreviations

RCT: randomized controlled trial
SG: serious game
