# Supplementary material for: Involvement of End Users in the Development of Serious Games for Health Care Professions Education: Systematic Descriptive Review
Source: JMIR Serious Games. 2021 Aug 19;9(3):e28650. doi: 10.2196/28650 (PMC8414295; doi:10.2196/28650)
Supplement: Multimedia Appendix 1 [file games_v9i3e28650_app1.docx]

CINAHL

1. TI ("serious gam*" OR "applied gam*" OR ((simulation OR training OR teaching OR educational OR education OR learning OR interactive) AND (((online OR electronic OR digital OR "overthecounter" OR commercial OR computer OR virtual OR "mobile application*" OR "mobile app") AND (game OR games OR gamification OR gaming)) OR (videogame* OR "video game*")))) OR AB ("serious gam*" OR "applied gam*" OR ((simulation OR training OR teaching OR educational OR education OR learning OR interactive) AND (((online OR electronic OR digital OR "over-the-counter" OR commercial OR computer OR virtual OR "mobile application*" OR "mobile app") AND (game OR games OR gamification OR gaming)) OR (videogame* OR "video game*"))))

2. (MH "Video Games+")

3. 1 OR 2

4. TI ("Health Personnel" OR "Health professional*" OR "Health care profession*" OR "Healthcare profession*" OR "Medical student*" OR "Medical assistant*" OR "health worker*" OR Audiologist* OR Chiropractor* OR Dentist* OR Dietitian* OR Dermatolog* OR End ocrinologist* OR Gastroenterolog* OR Gynecolog* OR Radiolog* OR "Medical Staff" OR Midwife* OR neurologi* OR nutritionist* OR Nurse* OR nursing OR Optometrist* OR "Occupational Therapist*" OR Patholog* OR Paramedic* OR Paediatric* OR pediatrician* OR Paediatrician* OR pediatric* OR Pharmacist* OR Pharmaconomist* OR Pharmacologist* OR "Pharmacy technician*" OR Phlebotomist* OR Physician* OR Podiatrist* OR Psychologist* OR Psychotherapist* OR psychiatrist* OR "Physical therapist*" OR physiotherapist* OR "Respiratory therapist*" OR Surgeon* OR surgical OR Clinician* OR Cardiologist* OR "medical technician*" OR "emergency doctor*" OR emergentologist* OR "clinical officer*" OR "Community health worker*" OR Radiographer* OR technologist* OR Radiotherapist* OR Anesthetist* OR Resident* OR trainee* OR intern*) OR AB ("Health Personnel" OR "Health professional*" OR "Health care profession*" OR "Healthcare profession*" OR "Medical student*" OR "Medical assistant*" OR "health worker*" OR Audiologist* OR Chiropractor* OR Dentist* OR Dietitian* OR Dermatolog* OR endocrinologist* OR Gastroenterolog* OR Gynecolog* OR Radiolog* OR "Medical Staff" OR Midwife* OR neurologi* OR nutritionist* OR Nurse* OR nursing OR Optometrist* OR "Occupational Therapist*" OR Patholog* OR Paramedic* OR Paediatric* OR pediatrician* OR Paediatrician* OR pediatric* OR Pharmacist* OR Pharmaconomist* OR Pharmacologist* OR "Pharmacy technician*" OR Phlebotomist* OR Physician* OR Podiatrist* OR Psychologist* OR Psychotherapist* OR psychiatrist* OR "Physical therapist*" OR physiotherapist* OR "Respiratory therapist*" OR Surgeon* OR surgical OR Clinician* OR Cardiologist* OR "medical technician*" OR "emergency doctor*" OR emergentologist* OR "clinical officer*" OR "Community health worker*" OR Radiographer* OR technologist* OR Radiotherapist* OR Anesthetist* OR Resident* OR trainee* OR intern*)

5. (MH "Health Personnel+") OR (MH "Students, Medical") OR (MH "Students, Nursing+")

6. (MH "Education, Premedical") OR (MH "Education, Medical+") OR (MH "Education, Nursing+") OR (MH "Education, Pharmacy")

7. 4 OR 5 OR 6

8. TI (Knowledge* OR Aptitude* OR accuracy OR abilit* OR capacity* OR confidence OR competenc* OR impact* OR skill* OR performance* OR "Learning outcome*" OR "training outcome*" OR effectiveness OR efficacy OR improvement* OR innovat* OR retention OR "randomi?ed controlled trial") OR AB (Knowledge* OR Aptitude* OR accuracy OR abilit* OR capacity* OR confidence OR competenc* OR impact* OR skill* OR performance* OR "Learning outcome*" OR "training outcome*" OR effectiveness OR efficacy OR improvement* OR innovat* OR retention OR "randomi?ed controlled trial")

9. (MH "Knowledge+") OR (MH "Clinical Competence+") OR (MH "Quality Improvement+") OR(MH "Learning+") OR (MH "Educational Measurement+") OR (PT "randomized controlled trial")

10. 8 OR 9

11. 3 AND 7 AND 10

12. 11 AND LA ( (english OR french) ) AND DT 20000101-20171231 AND PT Journal Article

**EMBASE (OVID)**

1. ("serious gam*" OR "applied gam*" OR ((simulation OR training OR teaching OR educational

OR education OR learning OR interactive) AND (((online OR electronic OR digital OR "overthecounter" OR commercial OR computer OR virtual OR "mobile application*" OR "mobile app") AND (game OR games OR gamification OR gaming)) OR (videogame* OR "video game*")))).ti,ab.

*2.* exp video game/

3. 1 OR 2

4. ("Health Personnel" OR "Health professional$1" OR "Health care profession*" OR "Healthcare profession*" OR "Medical student$1" OR "Medical assistant$1" OR "health worker$1" OR Audiologist$1 OR Chiropractor$1 OR Dentist$1 OR Dietitian$1 OR Dermatolog* OR endocrinologist$1 OR Gastroenterolog* OR Gynecolog* OR Radiolog* OR "Medical Staff" OR Midwife$1 OR neurologi*OR nutritionist$1 OR Nurse$1 OR nursing OR Optometrist$1 OR "Occupational Therapist$1" OR Patholog* OR Paramedic$1 OR Paediatric$1 OR pediatrician$1 OR Paediatrician$1 OR podiatrist$1 OR pediatric$1 OR Pharmacist$1 OR Pharmaconomist$1 OR Pharmacologist$1 OR "Pharmacy technician$1" OR Phlebotomist$1 OR Physician$1 OR Podiatrist$1 OR Psychologist$1 OR Psychotherapist$1 OR psychiatrist$1 OR "Physical therapist$1" OR physiotherapist$1 OR "Respiratory therapist$1" OR Surgeon$1 OR Surgical OR Clinician$1 OR Cardiologist$1 OR "medical technician$1" OR "emergency doctor$1" OR emergentologist$1 OR "clinical officer$1" OR "Community health worker$1" OR Radiographer$1 OR technologist$1 OR Radiotherapist$1 OR Anesthetist$1 OR Resident$1 OR trainee$1 OR intern$1).ti,ab.

5. exp health care personnel/ OR exp premedical student/ OR exp medical student/ OR exp nursing student/

6. exp medical education/ OR exp nursing education/ OR exp clinical education/

7. 4 OR 5 OR 6

8. (Knowledge$1 OR Aptitude$1 OR accuracy OR abilit* OR capacity* OR confidence OR competenc*OR impact$1 OR skill$1 OR performance$1 OR "Learning outcome$1" OR "training outcome*" OR effectiveness OR efficacy OR improvement$1 OR innovat* OR retention OR

"randomi?ed controlled trial").ti,ab.

9. exp clinical competence/ OR *total quality management/ OR exp learning curve/ OR exp knowledge/ OR exp Randomized Controlled Trials as Topic/

10. 8 OR 9

11. 3 AND 7 AND 10

12. 2000:2017.dp. AND (english OR french).la. AND Journal: Article.pt.

13. 11 AND 12

**ERIC (ProQuest)**

1. TI,AB("serious gam*" OR "applied gam*" OR ((simulation OR training OR teaching OR educational OR education OR learning OR interactive) AND (((online OR electronic OR digital OR "over-the-counter" OR commercial OR computer OR virtual OR "mobile application*" OR "mobile app") AND (game OR games OR gamification OR gaming)) OR (videogame* OR "video game*"))))

2. SU.EXACT("Video Games")

3. 1 OR 2

4. TI,AB("Health Personnel" OR "Health professional$1" OR "Health care profession*" OR "Healthcare profession*" OR "Medical student$1" OR "Medical assistant$1" OR "health worker$1" OR Audiologist$1 OR Chiropractor$1 OR Dentist$1 OR Dietitian$1 OR Dermatolog* OR endocrinologist$1 OR Gastroenterolog* OR Gynecolog* OR Radiolog* OR "Medical Staff" OR Midwife$1 OR neurologi*OR nutritionist$1 OR Nurse$1 OR nursing OR Optometrist$1 OR "Occupational Therapist$1" OR Patholog* OR Paramedic$1 OR Paediatric$1 OR pediatrician$1 OR Paediatrician$1 OR podiatrist$1 OR pediatric$1 OR Pharmacist$1 OR Pharmaconomist$1 OR Pharmacologist$1 OR "Pharmacy technician$1" OR Phlebotomist$1 OR Physician$1 OR Podiatrist$1 OR Psychologist$1 OR Psychotherapist$1 OR psychiatrist$1 OR "Physical therapist$1" OR physiotherapist$1 OR "Respiratory therapist$1" OR Surgeon$1 OR Surgical OR Clinician$1 OR Cardiologist$1 OR "medical technician$1" OR "emergency doctor$1" OR emergentologist$1 OR "clinical officer$1" OR "Community health worker$1" OR Radiographer$1 OR technologist$1 OR Radiotherapist$1 OR Anesthetist$1 OR Resident$1 OR OR trainee$1 OR intern$1)

5. SU.EXACT.EXPLODE("Health Personnel") OR SU.EXACT("Premedical Students")OR SU.EXACT.EXPLODE("Medical Students") OR SU.EXACT.EXPLODE("Nursing Students")

6. SU.EXACT.EXPLODE("Pharmaceutical Education") OR SU.EXACT.EXPLODE("Medical Education") OR SU.EXACT.EXPLODE("Nursing Education") OR SU.EXACT.EXPLODE("Clinical Experience")

7. 4 OR 5 OR 6

8. TI,AB(Knowledge$1 OR Aptitude$1 OR accuracy OR abilit* OR capacity* OR confidence OR competenc*OR impact$1 OR skill$1 OR performance$1 OR "Learning outcome$1" OR "training outcome*" OR effectiveness OR efficacy OR improvement$1 OR innovat* OR retention OR "randomi?ed controlled trial")

9. SU.EXACT.EXPLODE("Learning Processes") OR SU.EXACT.EXPLODE("Knowledge Level") OR SU.EXACT.EXPLODE("Skill Development") OR SU.EXACT.EXPLODE("Outcomes of Education")

10. 8 OR 9

11. 3 AND 7 AND 10

12. PD(2000-2017) AND LA(english OR french) AND DTYPE( journal articles)

13. 11 AND 12

**PsychINFO (APA PsychNet)**

1. **Title** : ("serious gam*" OR "applied gam*" OR ((simulation OR training OR teaching OR educational OR education OR learning OR interactive) AND (((online OR electronic OR digital OR "over-the-counter" OR commercial OR computer OR virtual OR "mobile application*" OR "mobile app") AND (game OR games OR gamification OR gaming)) OR (videogame* OR "video game*")))) *OR* **Abstract** : ("serious gam*" OR "applied gam*" OR ((simulation OR training OR teaching OR educational OR education OR learning OR interactive) AND (((online OR electronic OR digital OR "over-the-counter" OR commercial OR computer OR virtual OR "mobile application*" OR "mobile app") AND (game OR games OR gamification OR gaming)) OR (videogame* OR "video game*")))) *OR* **Index terms**: {Computer Games}

2. **Title** : ("Health Personnel" OR "Health professional*" OR "Health care profession*" OR "Healthcare profession*" OR "Medical student*" OR "Medical assistant*" OR "health worker*" OR Audiologist* OR Chiropractor* OR Dentist* OR Dietitian* OR Dermatolog* OR endocrinologist* OR Gastroenterolog* OR Gynecolog* OR Radiolog* OR "Medical Staff" OR Midwife* OR neurologi* OR nutritionist* OR Nurse* OR nursing OR Optometrist* OR "Occupational Therapist*" OR Patholog* OR Paramedic* OR Paediatric* OR pediatrician* OR Paediatrician* OR pediatric* OR Pharmacist* OR Pharmaconomist* OR Pharmacologist* OR "Pharmacy technician*" OR Phlebotomist* OR Physician* OR Podiatrist* OR Psychologist* OR Psychotherapist* OR psychiatrist* OR "Physical therapist*" OR physiotherapist* OR "Respiratory therapist*" OR Surgeon* OR surgical OR Clinician* OR Cardiologist* OR "medical technician*" OR "emergency doctor*" OR emergentologist* OR "clinical officer*" OR "Community health worker*" OR Radiographer* OR technologist* OR Radiotherapist* OR Anesthetist* OR Resident* OR trainee* OR intern*) *OR* **Abstract:** ("Health Personnel" OR "Health professional*" OR "Health care profession*" OR "Healthcare profession*" OR "Medical student*" OR "Medical assistant*" OR "health worker*" OR Audiologist* OR Chiropractor* OR Dentist* OR Dietitian* OR Dermatolog* OR endocrinologist* OR Gastroenterolog* OR Gynecolog* OR Radiolog* OR "Medical Staff" OR Midwife* OR neurologi* OR nutritionist* OR Nurse* OR nursing OR Optometrist* OR "Occupational Therapist*" OR Patholog* OR Paramedic* OR Paediatric* OR pediatrician* OR Paediatrician* OR pediatric* OR Pharmacist* OR Pharmaconomist* OR Pharmacologist* OR "Pharmacy technician*" OR Phlebotomist* OR Physician* OR Podiatrist* OR Psychologist* OR Psychotherapist* OR psychiatrist* OR "Physical therapist*" OR physiotherapist* OR "Respiratory therapist*" OR Surgeon* OR surgical OR Clinician* OR Cardiologist* OR "medical technician*" OR "emergency doctor*" OR emergentologist* OR "clinical officer*" OR "Community health worker*" OR Radiographer* OR technologist* OR Radiotherapist* OR Anesthetist* OR Resident* OR trainee* OR intern*) *OR* **Index terms**: {Allied Health Personne} OR {Health Personne} OR {Medical Personnel} OR {Mental Health Personnel} OR {Medical Students{ OR {Nursing Students} *OR* **Index terms**: {Medical Education} OR {Nursing Education} OR {Medical Internship} OR {Medical Residency} OR {Psychiatric Training}

3. **Title** : (Knowledge* OR Aptitude* OR accuracy OR abilit* OR capacity* OR confidence OR competenc* OR impact* OR skill* OR performance* OR "Learning outcome*" OR "training outcome*" OR effectiveness OR efficacy OR improvement* OR innovat* OR retention OR "randomi?ed controlled trial") *OR* **Abstract** : (Knowledge* OR Aptitude* OR accuracy OR abilit* OR capacity* OR confidence OR competenc* OR impact* OR skill* OR performance* OR "Learning outcome*" OR "training outcome*" OR effectiveness OR efficacy OR improvement* OR innovat* OR retention OR "randomi?ed controlled trial") *OR* **Index Terms:** {Declarative Knowledge} OR {Health Knowledge} OR {Job Knowledge} OR {Knowledge (General)} OR {Learning} OR {Procedural Knowledge} OR {Professional Competence} OR {Skill Learning} OR {Educational Measurement}

4. 1 AND 2 AND 3

5. **Language**:(english OR french) AND **Document type**: Journal Article AND **Year**: 2000 to 2017

6. 4 AND 5

PubMed

1. serious gam*[TIAB] OR applied gam*[TIAB] OR ((simulation[TIAB] OR training [TIAB] OR teaching[TIAB] OR educational[TIAB] OR education[TIAB] OR learning[TIAB] OR interactive[TIAB]) AND (((online[TIAB] OR electronic[TIAB] OR digital[TIAB] OR "over-the-counter"[TIAB] OR commercial[TIAB] OR computer[TIAB OR virtual[TIAB] OR mobile application*[TIAB] OR mobile app[TIAB]) AND (game[TIAB] OR games[TIAB] OR gamification[TIAB] OR gaming[TIAB] OR game-based[TIAB])) OR (videogame*[TIAB] OR video game*[TIAB)))
2. "Video Games"[MH]
3. #1 OR #2
4. Health Personnel*[TIAB] OR Health professional*[TIAB] OR Health care profession*[TIAB] OR Healthcare profession*[TIAB] OR Medical student*[TIAB] OR Medical assistant*[TIAB] OR health worker*[TIAB] OR Audiologist*[TIAB] OR Chiropractor*[TIAB] OR Dentist[TIAB] OR Dentists[TIAB] OR Dietitian*[TIAB] OR Dermatolog*[TIAB] OR endocrinologist*[TIAB] OR Gastroenterolog*[TIAB]OR Gynecolog*[TIAB]OR Radiolog*[TIAB] OR Medical Staff[TIAB] OR Midwife*[TIAB] OR neurologi*[TIAB] OR nutritionist*[TIAB] OR Nurse[TIAB] OR Nurses[TIAB] OR nursing[TIAB] OR Optometrist*[TIAB] OR Occupational Therapist*[TIAB] OR Patholog*[TIAB] OR Paramedic[TIAB] OR Paediatric[TIAB] OR pediatrician*[TIAB] OR Paediatrician*[TIAB] OR pediatrist*[TIAB] OR pediatric[TIAB] OR Pharmacist*[TIAB] OR Pharmaconomist*[TIAB] OR Pharmacologist*[TIAB] OR Pharmacy technician*[TIAB] OR Phlebotomist*[TIAB] OR Physician OR Podiatrist*[TIAB] OR Psychologist*[TIAB] OR Psychotherapist*[TIAB] OR psychiatrist*[TIAB] OR Physical therapist*[TIAB] OR physiotherapist*[TIAB] OR Respiratory therapist*[TIAB] OR Surgeon*[TIAB] OR surgical [TIAB] OR Clinician*[TIAB] OR Cardiologist*[TIAB] OR medical technician*[TIAB] OR emergency doctor*[TIAB] OR emergentologist*[TIAB] OR clinical officer*[TIAB] OR Community health worker*[TIAB] OR Radiographer*[TIAB] OR Radiotherapist*[TIAB] OR technologist[TIAB] Anesthetist*[TIAB] OR Resident[TIAB] OR residents[TIAB] OR trainee[TIAB] OR trainees[TIAB] OR intern[TIAB] OR interns[TIAB]
5. "Health Personnel"[MH] OR "Students, Premedical"[MH] OR "Students, Medical"[MH] OR "Students, Nursing"[Mesh]
6. "Education, Premedical"[MH] OR "Education, Medical"[MH] OR "Education, Nursing"[MH] OR "Education, Pharmacy"[MH] OR "Education, Public Health Professional"[MH] OR "Clinical Clerkship"[MH]
7. #4 OR #5 OR 6
8. knowledge*[TIAB] OR aptitude*[TIAB] OR accuracy[TIAB] OR ability[TIAB] OR abilities[TIAB] OR capacity [TIAB] OR capacities[TIAB] OR confidence[TIAB] OR compentency[TIAB] OR competencies[TIAB] OR impact*[TIAB] OR skill*[TIAB] OR performance*[TIAB] OR learning outcome*[TIAB] OR training outcome*[TIAB] OR effectiveness[TIAB] OR efficacy[TIAB] OR improvement*[TIAB] OR innovative*[TIAB] OR innovation*[TIAB] OR retention[TIAB] OR randomised controlled trial[TIAB] OR randomized controlled trial[TIAB]
9. "Clinical Competence"[MH] "Quality Improvement"[MH] OR "Learning Curve"[MH] OR Knowledge [MH] OR "Educational Measurement"[MH] OR "randomized controlled trial"[PT]
10. #8 OR 9
11. #3 AND #7 AND #10
12. (english[LA] OR french[LA]) AND 2000:2017[DP]
13. #11 AND #12

**Web of Sciences**

Science Citation Index Expanded (SCI-EXPANDED) --1945-present

Social Sciences Citation Index (SSCI) --1956-present

1. TS=("serious gam*" OR "applied gam*" OR ((simulation OR training OR teaching OR educational OR education OR learning OR interactive) AND (((online OR electronic OR digital OR "over-the-counter" OR commercial OR computer OR virtual OR "mobile application*" OR "mobile app") AND (game OR games OR gamification OR gaming)) OR (videogame* OR "video game*"))))

2. TS= ("Health Personnel" OR "Health professional$" OR "Health care profession*" OR "Healthcare profession*" OR "Medical student$" OR "Medical assistant$" OR "health worker$" OR Audiologist$ OR Chiropractor$ OR Dentist$ OR Dietitian$ OR Dermatolog* OR endocrinologist$ OR Gastroenterolog* OR Gynecolog* OR Radiolog* OR "Medical Staff" OR Midwife$ OR neurologi* OR nutritionist$ OR Nurse$ OR nursing OR Optometrist$ OR "Occupational Therapist$" OR Patholog* OR Paramedic$ OR Paediatric$ OR pediatrician$ OR Paediatrician$ OR pediatric$ OR Pharmacist$ OR Pharmaconomist$ OR Pharmacologist$ OR "Pharmacy technician$" OR Phlebotomist$ OR Physician$ OR Podiatrist$ OR Psychologist$ OR Psychotherapist$ OR psychiatrist$ OR "Physical therapist$" OR physiotherapist$ OR "Respiratory therapist$" OR Surgeon$ OR surgical OR Clinician$ OR Cardiologist$ OR "medical technician$" OR "emergency doctor$" OR emergentologist$ OR "clinical officer$" OR "Community health worker$" OR Radiographer$ OR technologist$ OR Radiotherapist$ OR Anesthetist$ OR Resident$ OR trainee$ OR intern$)

3. TS=("Clinical Clerkship" OR ((Clinical OR medical OR premedical OR pharma* OR nurse$) NEAR/3 (education OR training))

4. 2 OR 3

5. TS=(Knowledge$ OR Aptitude$ OR accuracy OR abilit* OR capacity* OR confidence OR competenc* OR impact$ OR skill$ OR performance$ OR "Learning outcome$" OR "training outcome*" OR effectiveness OR efficacy OR improvement$ OR innovat* OR retention OR "randomi?ed controlled trial")

6. 1 AND 2 AND 4

7. (PY=(2000-2017)) AND LANGUAGE: (English OR French) AND DOCUMENT TYPES: (Article OR Review)

8. 6 AND 7
