# Supplementary material for: Involvement of End Users in the Development of Serious Games for Health Care Professions Education: Systematic Descriptive Review
Source: JMIR Serious Games. 2021 Aug 19;9(3):e28650. doi: 10.2196/28650 (PMC8414295; doi:10.2196/28650)
Supplement: Multimedia Appendix 2 [file games_v9i3e28650_app2.docx]

**Supplementary Table 1. Key elements of end-user involvement in the development of serious games in healthcare professions education.**

| ***Name of the serious game*, author(s), year(s)** | **Role** | **Selection** | **Methods to elicit input** | **Elements for which input was elicited** | **Integration of input** |
| --- | --- | --- | --- | --- | --- |
| *Life Support Simulation Activities (LISSA)*  Boada et al. (2015); Wattanasoontorn et al. ; (Wattanasoontorn et al., 2014) | Testers | 30 nursing students | NR | Functional aesthetics | NR |
| *Uro-Island*  Boeker et al. (2009); Boeker et al. (2013) | Testers | 14 students from different medical schools | Self-reported questionnaires and written comments after playing the SG for the first time | Functional aesthetics,  Secondary characters,  Goals | NR |
| *Delirium Experience*  Buijs-Spanjers et al. (2018); Buijs-Spanjers et al. (2019) | Testers | NR | NR | Functional aesthetics | Input was integrated but no details given. |
| *Play-learn inhalation game*  Chee et al. (2019) | Co-designers | NR | NR | Goals | NR |
| *PULSE*  Cook et al. (2012) | Feedback and answers to specific concerns | NR | Focus groups | NR | NR |
| *Tic-Tac-Toe*  Courtier et al. (2016) | Testers | NR | NR | NR | Input was integrated but no details given. |
| *Air Medic Sky-1*  Dankbaar et al. (2017); Huiskes et al. (2015) | Testers | 27 sixth-year medical students | Self-reported questionnaires and written comments after free use of the SG during an unspecified period | Goals  Functional aesthetics | Input was integrated but no details given. |
| *Operating Theater Game*  del Blanco et al. (2013); Del Blanco et al. (2017) | Testers | Undisclosed number of final-year nursing and medical students | Undescribed Likert scales. | Feedback | Input was integrated but no details given. |
| *InsuOnline*  Diehl, de Souza, et al. (2015); Diehl, Gordan, et al. (2015); Diehl et al. (2013); Diehl et al. (2017) | Testers | 1) 6 undergraduate medical students and physicians  2) 20 undergraduate medical students  Equal number of women and men with varying degrees of gaming experience | During a single session of play in which the third of the serious game was available:  1) Two self-reported questionnaires;  2) Recording of end-user actions in the serious game;  3) Think aloud. | Functional aesthetics  Hedonic aesthetics  Goals  Feedback | Addition of a progression bar to provide feedback on progression.  Emphasis on visual cues in the virtual environment.  Reduction of the amount of onscreen written material. Additions of highlights and shadows to facilitate cursor visualization. Correction of technical glitches. Addition of an option to turn down the background music. |
| *The Medication Game*  Foss et al. (2014); Foss et al. (2013); Mordt et al. (2011) | 1) Testers  2) Consulted about their needs or preferences | NR | Undescribed questionnaires | Hedonic aesthetics  Goals | Adjustment of the level of challenge to learners’ abilities.  Addition of an option to turn off the background music. |
| *Dr. Game, Surgeon Trouble*  Graafland et al. (2014, 2017) | Feedback and answers to specific concerns | NR | NR | Goals | NR |
| *SG name NR*  Kerfoot et Baker (2012); Kerfoot et al. (2009) | Feedback and answers to specific concerns | 32 urologists | End-users were asked to answer the questions that were to be integrated to the serious game | Goals | Adjustment of the level of challenge to learners’ abilities. |
| *Triage Trainer*  Jarvis et Freitas (2009); Knight et al. (2010) | Testers | 12 various healthcare workers with knowledge of the medical management major incidents | NR | Feedback | NR |
| *Hygie*  Jaunay et al. (2019) | Testers | 20 general practitioners or residents | NR | NR | Adjustment of the level of challenge to learners’ abilities.  Correction of technical glitches |
| *Night Shift*  Mohan et al. (2017); Mohan et al. (2016) | Reported to be involved but role could not be determined | NR | NR | NR | NR |
| *Shift: The Next Generation*  Mohan et al. (2018) | 1) Testers  2) Feedback and answers to specific concerns | 36 emergency medicine physicians | NR | Functional aesthetics | NR |
| *Resus Days*  *Phungoen et al. (2020)*  *Panyowat (2019)*  *Panyowat (2018)* | 1. Consulted about their needs or preferences; 2. Feedback and answers to specific concerns 3. Testers | Undisclosed number of physicians | NR | Functional aesthetics  Goals | Input was integrated but no details given. |
| *HH-VSTS*  Polivka et al. (2019) | Reported to be involved but role could not be determined | NR | NR | NR | NR |
| *SG name NR*  Scales et al. (2016) | Feedback and answers to specific concerns | 34 residents and fellows | End-users were asked to answer the questions that were to be integrated to the serious game | Goals | NR |
| *SG name NR*  Tan et al. (2017) | Reported to be involved but role could not be determined | NR | NR | Goals | Adjustment of the level of challenge to learners’ abilities. |
| *Online Competitive Anatomy Tournament*  Van Nuland et al. (2015) | Reported to be involved but role could not be determined | NR | NR | NR | NR |

Note. NR: non-reported
